# The Ussing chamber system for measuring intestinal permeability in health and disease

**DOI:** 10.1186/s12876-019-1002-4

**Published:** 2019-06-20

**Authors:** Amanda Thomson, Kathryn Smart, Michelle S. Somerville, Sarah N. Lauder, Gautham Appanna, James Horwood, Lawrence Sunder Raj, Brijesh Srivastava, Dharmaraj Durai, Martin J. Scurr, Åsa V. Keita, Awen M. Gallimore, Andrew Godkin

**Affiliations:** 1 Institute of Infection and Immunity, School of Medicine, Cardiff, UK; 2 Department of Gastroenterology and Hepatology, University Hospital Wales, Cardiff, UK; 3 Department of Surgery, University Hospital Wales, Cardiff, UK; 4 Division of Surgery, Orthopedics & Oncology, Department of Clinical and Experimental Medicine, Linköping University, Linköping, Sweden; 5 Present address: Institute of Cellular Medicine, Newcastle University, Newcastle Upon Tyne, UK

**Keywords:** Intestinal permeability, Colon, Transepithelial resistance, Paracellular flux

## Abstract

**Background:**

The relationship between intestinal epithelial integrity and the development of intestinal disease is of increasing interest. A reduction in mucosal integrity has been associated with ulcerative colitis, Crohn’s disease and potentially could have links with colorectal cancer development. The Ussing chamber system can be utilised as a valuable tool for measuring gut integrity. Here we describe step-by-step methodology required to measure intestinal permeability of both mouse and human colonic tissue samples ex vivo, using the latest equipment and software. This system can be modified to accommodate other tissues.

**Methods:**

An Ussing chamber was constructed and adapted to support both mouse and human tissue to measure intestinal permeability, using paracellular flux and electrical measurements. Two mouse models of intestinal inflammation (dextran sodium sulphate treatment and T regulatory cell depletion using C57BL/6-FoxP3^DTR^ mice) were used to validate the system along with human colonic biopsy samples.

**Results:**

Distinct regional differences in permeability were consistently identified within mouse and healthy human colon. In particular, mice showed increased permeability in the mid colonic region. In humans the left colon is more permeable than the right. Furthermore, inflammatory conditions induced chemically or due to autoimmunity reduced intestinal integrity, validating the use of the system.

**Conclusions:**

The Ussing chamber has been used for many years to measure barrier function. However, a clear and informative methods paper describing the setup of modern equipment and step-by-step procedure to measure mouse and human intestinal permeability isn’t available. The Ussing chamber system methodology we describe provides such detail to guide investigation of gut integrity.

**Electronic supplementary material:**

The online version of this article (10.1186/s12876-019-1002-4) contains supplementary material, which is available to authorized users.

## Background

The intestinal epithelial barrier separates luminal contents from the internal body, crucial for maintaining the symbiotic relationship between the host and intestinal microbes. It is composed of epithelial cells, linked together by tight junctional (TJ) protein complexes, required for sealing the paracellular space [1–3]. Impairment of barrier function has been linked to intestinal diseases such as ulcerative colitis and Crohn’s disease [4–7]. Gut integrity can be assessed in vivo*,* by measuring the presence of molecular probes such as sugars or dextran in urine or blood after oral intake [8, 9]. Whilst this method can determine overall differences in permeability between the small and large intestine, it is unable to reveal detailed information relating to precise regional locations of altered integrity within each organ and also lacks standardisation [9]. Additionally, external factors including gastrointestinal motility and mucosal blood flow may lead to inaccuracy of permeability measurements.

The eponymously named Ussing chamber takes its name from Danish biologist Hans Ussing, who developed the technique in the 1950s to understand the phenomenon of active Na^+^ transport [10]. Such seminal studies paved the way for the present models of transepithelial transport, including the discovery of the Na^+^/K^+^ ATPase pump [11, 12]. In later years, the Ussing system helped unravel the mechanistic processes underpinning cystic fibrosis (CF). By monitoring ionic movements it was shown that Cl^−^ secretion was decreased in patients [13–15], due to a mutated CFTR gene [16].

**Fig. 1:**
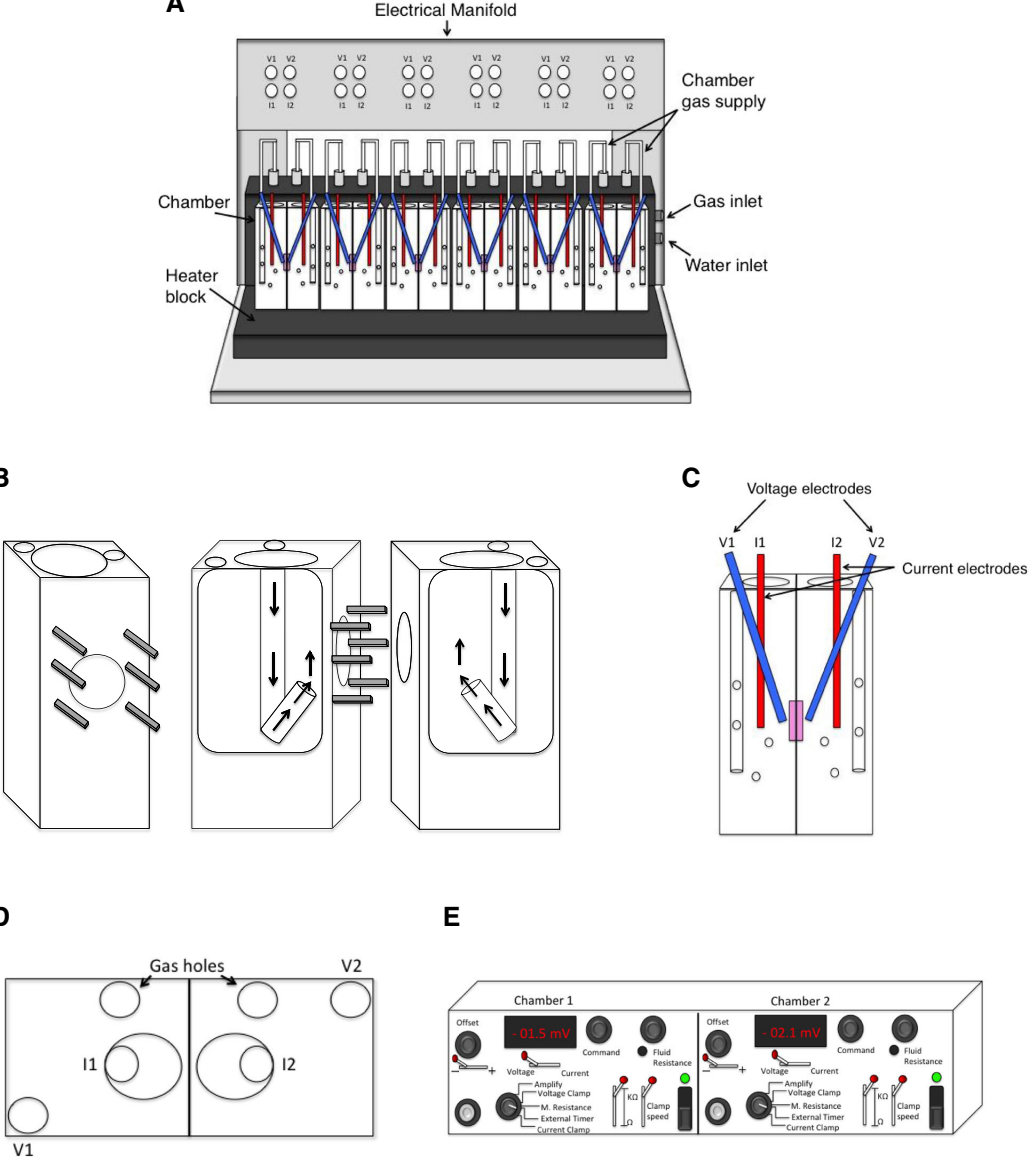
Design of the Ussing chamber system. **a** Navicyte Ussing system unit supporting six chambers with custom made electrical manifold. **b** Chamber halves showing metal pins used to secure tissue. Gas flow direction to stir buffer is depicted by arrows. **c** Individual Ussing chamber with voltage and current Ag/AgCl electrodes. **d** Birds-eye view of individual Ussing chamber showing electrode and gas inserts. **e** Amplifying EC825A voltage clamp box containing a volt and amp meter to record voltage and current respectively. External timer is selected to connect to computer software. Figures are original, created using Microsoft PowerPoint for Mac version 16.20

The Ussing system offers an ex vivo measurement of permeability using fluorescent probes as well as electrophysiological measurements. The system design has been updated to accommodate multiple chambers as shown in Fig. 1a. The chambers support epithelial tissue or cell monolayers in such a way that each side of the membrane is isolated and faces a separate chamber-half. The potential difference (PD) across epithelial tissue can be determined using Ag/AgCl electrodes, giving an indicator of tissue health [17]. Transepithelial resistance (TER) can be calculated to give an overall measurement of gut integrity. A low TER value is indicative of increased permeability. Short circuit current (Isc) is also often used during electrophysiological measurements. Isc refers to the current that is required to nullify tissue PD and is a summation of all ionic currents across the epithelium. Isc can be calculated from PD and TER values. Prior studies have shown that decreased TER under inflammatory conditions was associated with down regulation of “sealing” TJ proteins e.g. ZO-1 [18], JAM-A [19] and claudins-1 [20], − 3 and − 5 [21] as well as up-regulation of pore forming proteins (claudin-2 [22]). The use of fluorescently labelled probes such as 4 kDa FITC-dextran and lucifer yellow provide measurements of paracellular flux across the epithelium. We describe in depth the modern multi-chambered Ussing system, with complementary software protocol. Furthermore, we detail essential methods for dissecting and mounting tissue samples to ensure both viability and consistency of results. Methods are illustrated using colonic tissue from mouse models of intestinal inflammation and human samples.

## Methods

### Buffers

Krebs buffers are summarised in Table 1. All chemicals were purchased from Sigma Aldrich, UK. Transport Krebs, mannitol Krebs and glucose Krebs were used at pH 7.3, set after buffers were oxygenated.

**Table 1:** Krebs buffers and concentrations

Buffer	Concentration
Krebs Stock	136 mM NaCl, 1.5 mM CaCl_2_*2H_2_O, 4.3 mM KCl, 1.6 mM KH_2_PO_4_, 27 mM NaHCO_3_, 1.4 mM MgSO_4_*7H_2_
Glutamate Stock	114 mM Glutamate
Sodium Pyruvate Stock	115 mM Sodium Pyruvate
Glucose Stock	200 mM Glucose
Mannitol Stock	200 mM Mannitol
Transport Krebs	115.6 mM NaCl, 1.3 mM CaCl_2_*2H_2_O, 3.7 mM KCl, 1.4 mM KH_2_PO_4,_ 23 mM NaHCO_3_, 1.2 mM MgSO_4_*7H_2_
Glucose Krebs	5.8 mM Sodium Pyruvate, 5.7 mM Glutamate, 10 mM Glucose
Mannitol Krebs	5.8 mM Sodium Pyruvate, 5.7 mM Glutamate, 10 mM Mannitol

### Mice

C57BL/6-FoxP3^DTR^ mice gifted by Alexander Rudensky, described by Kim et al. [23] were housed in filter top cages at the specific pathogen free (SPF) Joint Biological Service Unit (Cardiff University, UK). All experiments were performed in the home cage. Mice were maintained at a constant temperature, with 12-h light/dark cycle and had access to standard mouse chow and water *ad libitium*. All experiments performed were ethically approved by the UK Home Office, in accordance with the Animal Scientific Procedures Act 1986, in compliance with UK Home Office regulations and were carried out by individuals holding a UK personal home office licence. All mice were monitored daily and humanely sacrificed by exposure to a rising concentration of 2–4% CO_2_, before gut integrity was measured. The National Centre for the Replacement, Refinement and Reduction of Animals in Research (NC3Rs) handling guidelines were also followed within this study. An animal research: reporting of in-vivo experiments (ARRIVE) guidelines checklist for working with animals can be found in Additional file 2.

4 male and 4 female 13–16-week-old C57BL/6-FoxP3^DTR^ mice were used to demonstrate baseline measurements. Results are comprised of four individual experiments due to constraints relating to Ussing chamber size. To deplete T regulatory cells (Tregs), diphtheria toxin (DTx; Native Antigen, UK) in 300 μL PBS was administered by standard intraperitoneal (i.p.) injection (15 μg/kg body weight) to three female C57BL/6-FoxP3^DTR^ healthy 16-week-old mice, chosen at random, every other day for 15 days. Three age and sex-matched mice were used as untreated controls. Treated and untreated animals were housed in the same cage.

Medium weight dextran sodium sulphate (DSS) (MP Biomedicals, UK) is widely used as a model of intestinal disease [24]. 3% DSS was given to eight healthy 10-week-old Treg replete female C57BL/6-FoxP3^DTR^ mice, chosen at random, in drinking water for up to 9 days to induce acute inflammation. Individual mice were housed in separate cages to monitor the intake of drinking water. Four age and sex-matched mice were in the untreated control group. Results are comprised of five independent experiments due to the size of the Ussing chamber.

### Setup of chambers

The Navicyte heating block was used for all experiments. It accommodates six acrylic chambers and is connected to a circulating water bath (Harvard Apparatus, UK) (Fig. 1a) to maintain chambers at 37 °C. Chambers were cleaned with RBS buffer 25 (Sigma Aldrich, UK) in hot water using a small toothbrush and dried before use. Inner surfaces of chamber halves (Fig. 1b) were coated with a medium, even layer of vacuum grease (Sigma Aldrich, UK) using a cotton bud. X-ray film squares with the correct aperture size to accommodate the required tissue specimen were made using a hole punch and placed over chamber pins. Chamber halves were connected (Fig. 1b and c) and secured in place with circlips using circlip pliers (Harvard Apparatus, UK). 1.5 ml transport Krebs buffer was added to each side of the chamber.

### Setup of ag/AgCl electrodes

Electrodes arrived in a “ready to use” state, having been previously coated with AgCl (Harvard Apparatus, UK). It is essential to re-chloride electrodes to maintain stable measurements. After experiments, old AgCl coating was removed from electrodes by gently rubbing with steel wool. Electrodes were cleaned with dH_2_O and immersed in household bleach for 24–48 h before the beginning of experiments to ensure efficient chloriding of the silver wire. Electrodes were washed with dH_2_O before use. Note that poor chloriding of electrodes will result in drifting PD values during the initial setup, making it impossible to zero any offset potential before tissue collection.

3 M KCl saturated with Ag electrolyte solution (Sigma Aldrich, UK) was dispensed into a small bottle containing a holder for a thin plastic tube. The tube was inserted to the end of the electrode glass barrel (Harvard Apparatus, UK) where a ceramic junction is located. Using constant pressure, in order that electrolyte solution flows continually, the barrel is filled as the tube is slowly withdrawn. Tapping lightly against the ceramic junction dislodges micro air bubbles. KCl salt can form at the opening of the glass barrel. This was carefully removed to prevent blockages of the ceramic junction, which can lead to interference or obstruction of electrical readings. Note that air bubbles and blockage of the ceramic junction are the most common reason for poor electrical readings. Glass barrels should be replaced if necessary. Electrodes were assembled and positioned to be close to each other but not touching within the chamber (Fig. 1c and d). Current pulses were sent across the chamber using an EC825A volt/clamp box (Warner Instruments Inc., Connecticut, USA) (Fig. 1e) to measure the resistance created by the buffer and presence of x-ray film pieces (see Electrical measurements). During analysis the “buffer resistance” was subtracted from total resistance readings.

### Murine dissection technique and mounting

**Fig. 2:**
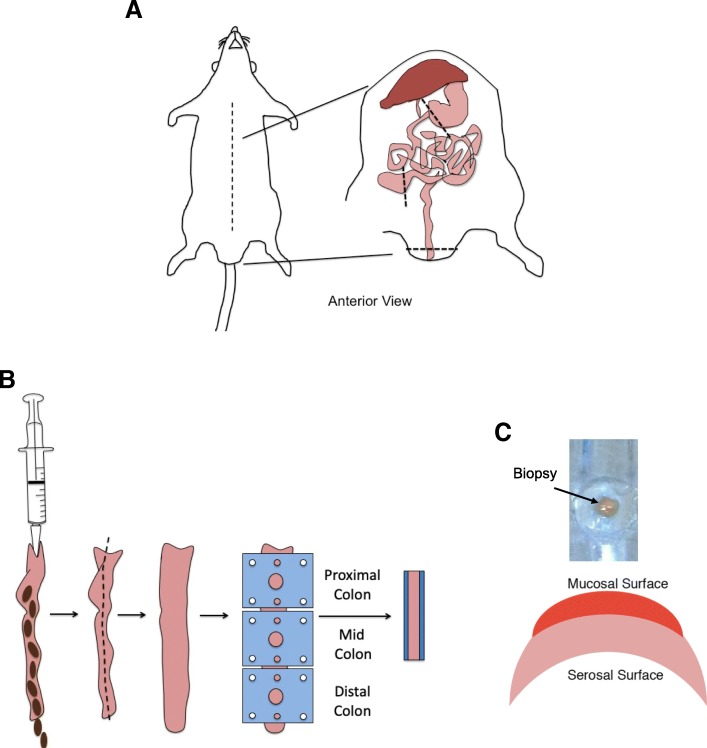
Mouse dissection and mounting technique. **a** Midline incision showing exposed abdominal organs. Black dotted lines indicate dissection points to obtain the intestine during the procedure. **b** Colon is flushed with Krebs buffer and opened longitudinally into a flat sheet using micro-dissecting scissors. X-ray film squares with the desired aperture are applied to the proximal, mid or distal regions. A second x-ray film with corresponding aperture size is placed on top, sandwiching tissue in-between. **c** Human biopsy sample showing contraction of serosal tissue leads to formation of a crescent morphology with the mucosal surface facing upwards. Figures are original, created using Microsoft PowerPoint for Mac version 16.20

A midline incision was made, and the colon dissected from the small intestine by cutting across the bottom half of the caecum (Fig. 2a). Faecal contents were removed by flushing with oxygenated transport Krebs buffer (Fig. 2b). The colon was pinned onto a cold sylgard plate (Ellsworth Adhesives, East Kilbride, Scotland) and opened longitudinally using watch-maker forceps and micro-dissection scissors (Fig. 2b). Desired colonic sections were selected with x-ray film squares that exhibit a 3 mm diameter hole and subsequently mounted onto chamber pins (Fig. 2b). Corresponding x-ray film squares were placed on top, sandwiching tissue in-between to prevent leakage. 1.5 ml cold oxygenated transport Krebs buffer was gently added to each chamber-half, avoiding dislodging the tissue. Chambers were placed on ice and transported to the laboratory within 10 minutes. Note that any damage to the mucosal surface will result in poor readings.

### Human biopsy collection and mounting

Colonic biopsies were obtained from 17 patients undergoing colonoscopy procedures at The University Hospital of Wales (patient characteristics are given in Additional file 1: Table S1). Prior to colonoscopy, patients consumed laxatives (Moviprep or Citramag and Senna), following Cardiff and Vale health board instructions. All subjects gave informed consent to take part in this study. The Wales Research Ethics Committee granted ethical approval (15/WA/0291). Approximately six biopsies from each patient were obtained using oval fenestrated non-spiked swing jaw disposable forceps (Olympus, UK). Note that spiked forceps would puncture tissue samples. Biopsies were immediately put into cold oxygenated transport Krebs buffer and transported to the laboratory within 10 minutes. Whilst remaining immersed in cold oxygenated transport Krebs buffer, biopsies were examined for damage under 5X magnification and carefully unfolded. The mucosal surface was identified as shown in Fig. 2c. Biopsies were mounted mucosal side up, in-between two pieces of x-ray film with an aperture size of 0.79 mm^2^_._ Chambers were closed and secured with circlips.

### Equilibration of tissue

Serosal chamber-halves were filled with 1.5 ml cold glucose Krebs buffer (10 mM) to provide tissue with an energy source. Mucosal halves were filled with 1.5 ml cold mannitol Krebs buffer (10 mM) to maintain osmotic balance without influencing glucose mediated Na^+^ transport. Electrodes were inserted as shown in Fig. 1c and d to monitor PD values. Chambers were warmed to 37 °C via the circulating water bath and continuously oxygenated with 95% O_2_, 5% CO_2_ to aid tissue viability and provide buffer circulation. Buffers at 37 °C were renewed after 20 minutes and again at 30 minutes to remove auto-fluorescent molecules before measurements began.

### Fluorescent probe addition

After equilibration, mannitol-krebs was replaced with 150 μl 4 kDa FITC-dextran (Sigma Aldrich, UK. Catalog number: 46944) during mouse studies or CH dilithium lucifer yellow during human studies (Sigma Aldrich, UK. Catalog number: L0259). Starting concentrations of 0.5 mg/ml or 1 mg/ml 4 kDa FITC-dextran were used. Initial experiments revealed that 4 kDa FITC-dextran was not easily detected within healthy mouse tissue. Therefore, the smaller probe lucifer yellow (457 Da) was chosen for human studies. Lucifer yellow was added at 250 μg/ml. Paired mucosal and serosal samples were obtained at 60 minutes and fluorescence analysed on the Clariostar plate reader (BMG, Germany). Serosal concentrations were determined using a standard curve generated from known concentrations. Paracellular flux is given as μg/ml/cm^2^.

### Electrical measurements

**Fig. 3:**
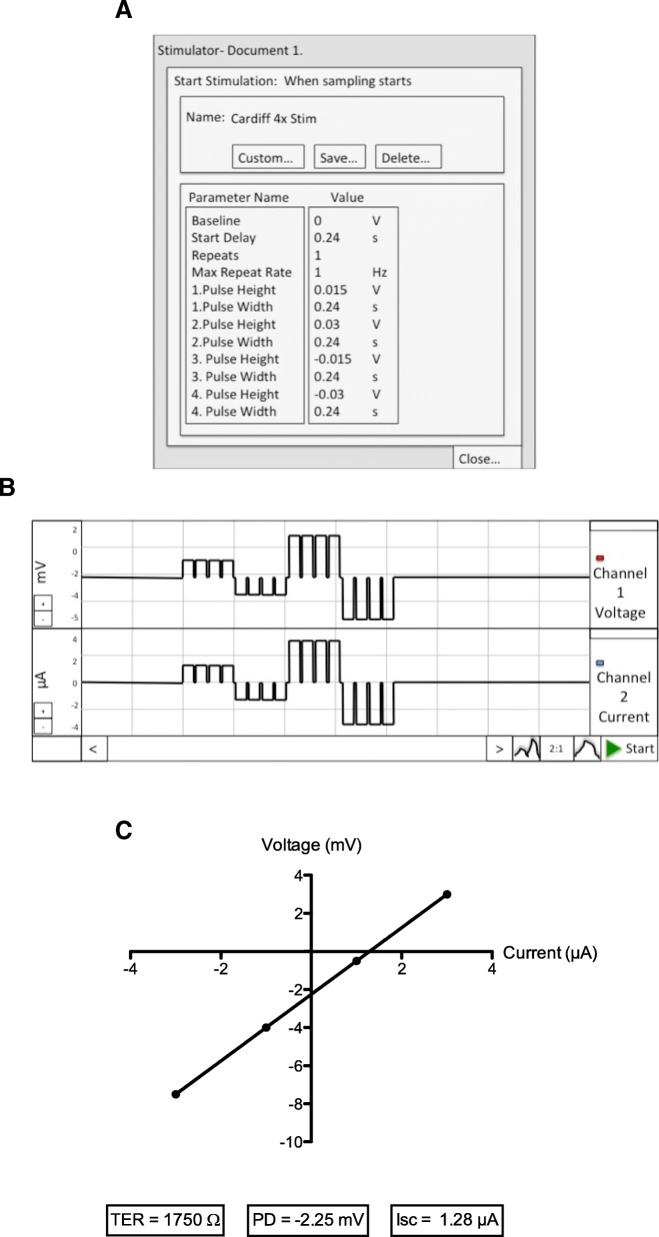
Electrical measurements. **a** Current pulse stimulation event protocol created in LabChart software. **b** Diagram showing current stimulation traces and tissue PD response. **c** Least fit squares analysis based on Ohms law. Current stimulation is plotted against tissue voltage. Linear regression is equal to TER. The y-intercept equals the PD value of tissue and Isc can be calculated by PD/TER

Spontaneous tissue potential difference (PD) was measured continuously throughout experiments via voltage Ag/AgCl electrodes. Experiments were performed in open circuit conditions where short current pulses of 1.5, − 1.5, 3 and -3 μA with a duration of 240 ms were sent across the tissue every 5 minutes via Ag/AgCl current passing electrodes (current clamping). Current stimulation was generated from an EC825A voltage/clamp unit (Fig. 1e), controlled by a LabChart software protocol developed in-house (Fig. 3a). The PD response to a fixed “clamped” current was measured at each stimulation event and mean voltage response of four recordings calculated (Fig. 3b). Tissue TER was calculated using a linear least squares fit analysis based on Ohms Law: resistance (R) = voltage (V) / current (I). TER is equal to the slope of the line generated and resting tissue PD equal to the intersection of the Y-axis. Isc was not directly monitored in this study but can be calculated by PD/TER. (Fig. 3c). TER is given as Ω (ohms)*cm^2^ by multiplying by the exposed tissue area. At 60 minutes, the cAMP-dependent Cl^−^ secretagogue forskolin (Sigma Aldrich, UK) was added to the serosal chamber at a final concentration of 6.6 μM to evaluate tissue viability. Only samples with a PD value of 0.5 mV or less and a response to forskolin, as shown by a drop in PD were included in analyses. A forskolin response can also be identified by an increase in Isc.

### Histological analysis

Mouse colonic tissue was wrapped around a large pair of forceps to create a “gut roll” and pinned in place with a 23G needle. Samples were fixed in 10% neutral buffered formalin solution (NBFS) (Sigma Aldrich, UK) for approximately 7 days then put into 70% ethanol. For human biopsy samples 10% NBFS was added directly to chambers upon completion of Ussing experiments for 2 days. Samples were removed and placed in universals containing 10% NBFS for a further 24 h before being transferred into 70% ethanol. Samples were agar embedded to ensure correct orientation of the tissue before being processed and embedded into paraffin wax blocks. 5 μm sections were cut and stained with Harris haematoxylin (Sigma Aldrich, UK) and eosin Y solution (Sigma Aldrich, UK).

### Statistical analysis

GraphPad Prism version 5 was used for all statistical analyses. Groups were assessed for normal distribution. A student’s T test or Mann Whitney test was applied to test for statistically significant differences. A *P*-value ≤0.05 was considered significant.

## Results

### Regional variation of intestinal integrity occurs within healthy mice

**Fig. 4:**
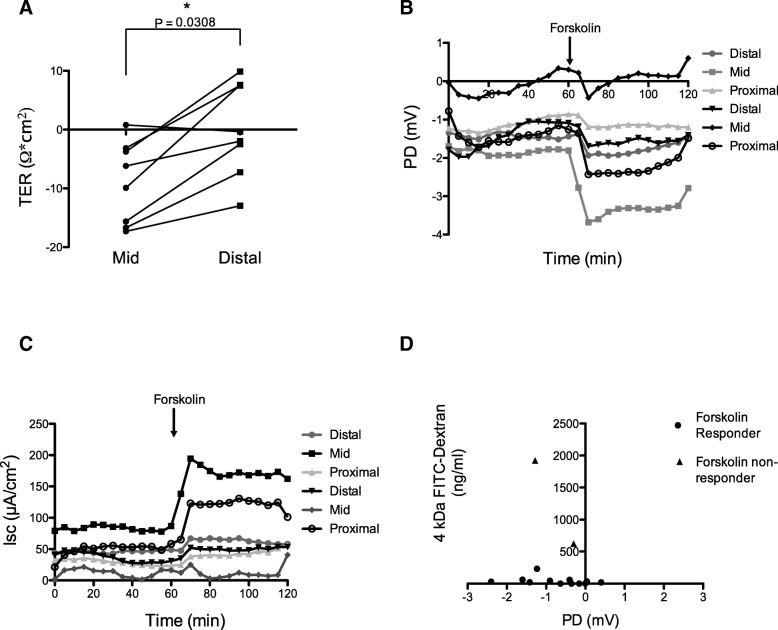
Baseline TER identifies regional permeability differences. **a** TER of mid and distal colonic regions normalised to the proximal colonic region from individual untreated FoxP3^DTR^ mice. **b** Negative PD values showing tissue responds to Forskolin at sixty minutes. **c** Isc values. **d** Tissue with a negative PD value shows little or no 4 kDa FITC-dextran in serosal chamber. Black triangles indicate tissue not responsive to forskolin, therefore removed from further analyses. Student’s unpaired t-test, statistical significance is indicated: *p = < 0.05. Error bars show +/− SEM

Regional variations of intestinal integrity are not well defined. Therefore the TER across proximal, mid and distal colonic sections from untreated FoxP3^DTR^ mice was investigated using the Ussing chamber system. Male and female mice show TER is lowest in the mid-section of the colon and highest in the proximal region (Fig. 4a), thus the mid-region of the colon is naturally most permeable. Comparing TER between male and female mice, there was no significant difference (Additional file 3: Figure S1A-S1C). A trend for lower TER in the mid colonic region of female mice was observed (Additional file 3: Figure S1B). Addition of forskolin to chambers resulted in a drop in PD (Fig. 4b) mirrored by an increase in Isc (Fig. 4c). Mucosal integrity was confirmed by lack of 4 kDa FITC-dextran passage (Fig. 4d).

### Measuring intestinal permeability in chemical-induced colitis

**Fig. 5:**
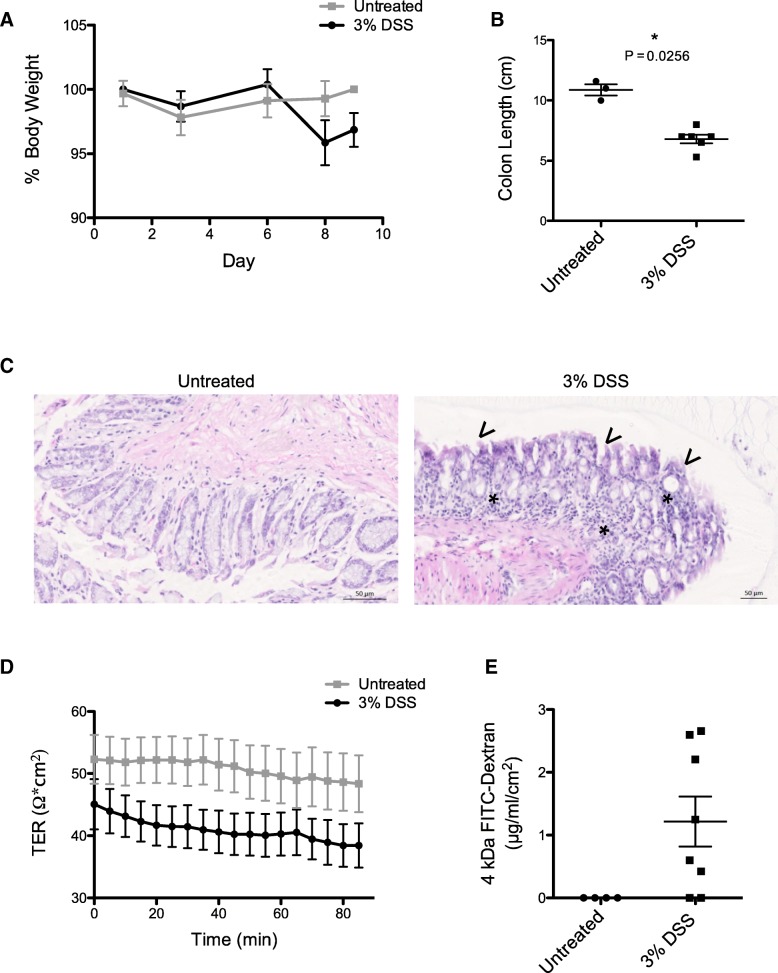
3% Dextran Sodium Sulphate causes intestinal inflammation and increased intestinal permeability. 10-week-old mice were administered 3% DSS within drinking water for up to nine days to induce acute intestinal inflammation. **a** Percentage body weight and (**b**) colon length of animals. **c** Representative haematoxylin and eosin immunohistochemistry staining of colonic tissue. Asterisks and arrows indicate increased immune cell infiltration and disrupted crypt structure respectively. **d** TER measurements of proximal colon from DSS treated and untreated animals. **e** 4 kDa FITC-dextran flux across colonic tissue. *n* = 4 untreated vs 8 treated. Error bars show +/− SEM

DSS induces intestinal inflammation (colitis) in mice [24]. Addition of 3% DSS to drinking water leads to reduction in mouse weight (Fig. 5a) and shortening of colon length (Fig. 5b) within 9 days. This was associated with disruption of normal epithelial intestinal architecture of the colon (Fig. 5c). Ussing chamber analysis of proximal colonic regions showed reduced TER (Fig. 5d) and increased paracellular flux of 4 kDa FITC-dextran (Fig. 5e). Together these data demonstrate increased intestinal permeability with DSS-induced colitis.

### Measuring intestinal permeability in auto-immune colitis

**Fig. 6:**
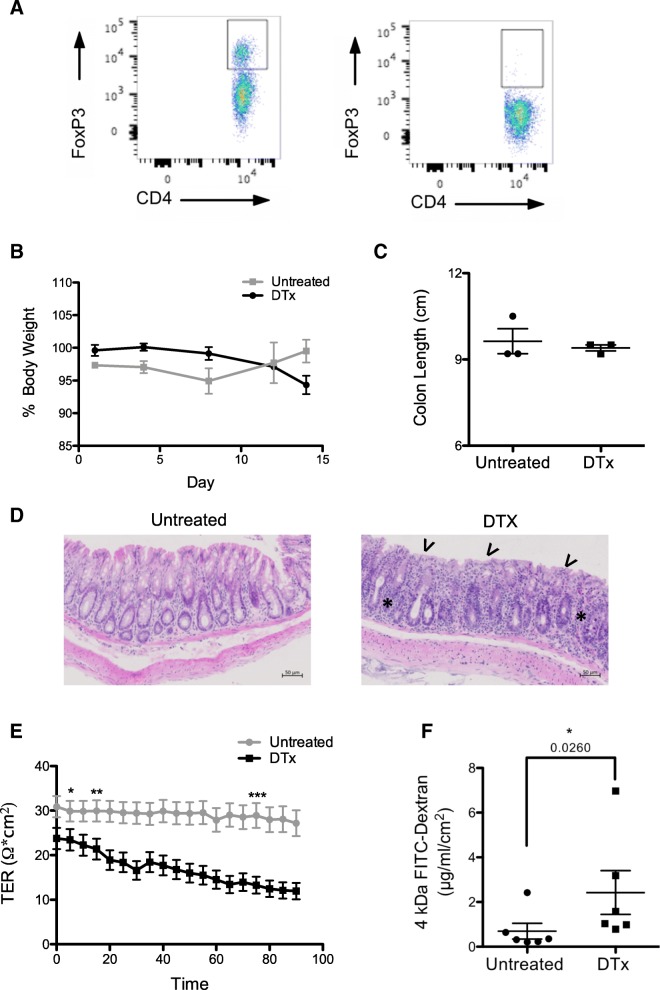
T-regulatory cell depleted mice (DTx) show increased intestinal permeability. **a** Representative FACS plots showing depletion of FoxP3^+^ cells. **b** Percentage body weight and (**c**) colon length of untreated and treated mice. **d** Haematoxylin and eosin staining of distal colonic sections from untreated and treated animals. Asterisks and arrows indicate increased immune cell infiltration and disrupted crypt structure respectively. **e** TER and (**f**) 4 kDa FITC-dextran passage of proximal and mid colonic sections. Mucosal starting concentration was 1 mg/ml. *n* = 3 treated vs 3 untreated 16-week-old female mice. Unpaired students T tests and a Mann Whitney test were used for comparisons in (**e**) and (**f**) respectively. Significant differences indicate: *P < 0.05, **P < 0.001, ***P < 0.001. Error bars show +/− SEM

Tregs are a major component of the immune system, responsible for controlling immune responses, reducing inflammation and promoting tolerance towards commensal microbiota and food antigens within the intestine [25–28]. Depletion of Tregs (Fig. 6a) causes the development of autoimmune symptoms, including colitis (weight loss and diarrhoea) [29]. Treg-depleted mice were sacrificed once signs of autoimmunity (piloerection, scaly skin, listless behavior) were evident and gut integrity assessed. In comparison to untreated controls, Treg-depleted mice exhibited weight loss by day 14 (Fig. 6b). This was not accompanied by shortening of colon length (Fig. 6c). Depleted mice had abnormal colonic architecture, indicative of inflammation (Fig. 6d), and displayed a reduction in TER (Fig. 6e). TER also declined rapidly, suggesting that removal of Tregs reduces the tissue’s ability to maintain efficient tight junctional structures under stress. The fall in TER was accompanied by increased 4 kDa FITC-dextran paracellular flux (Fig. 6f). Collectively, these data indicate that Tregs have a role in promoting gut integrity and maintaining intestinal homeostasis.

### Human biopsy intestinal permeability

**Fig. 7:**
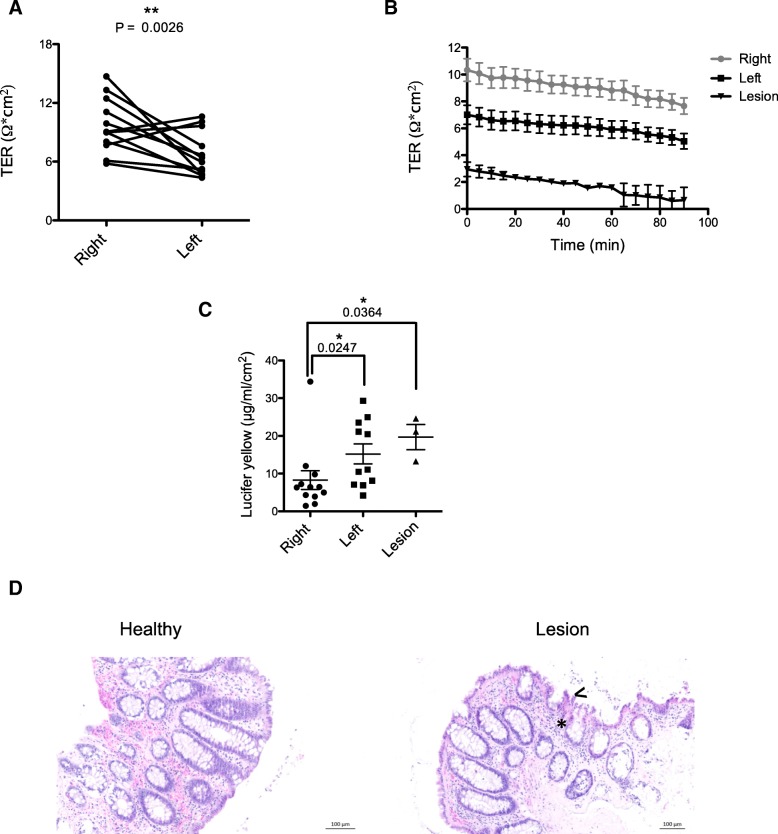
Human permeability measurements. **a** TER of paired right and left colon biopsies from control patients. **b** TER and (**c**) lucifer yellow passage of right and left colon biopsies taken from control patients or from a lesion site. **d** Haematoxylin and eosin staining of control tissue or tissue from a lesion site. Asterisks and arrows indicate increased immune cell infiltration and disrupted crypt structure respectively. Unpaired two-tailed students T-test and Mann-Whitney tests used for statistical analyses. Error bars show +/− SEM

Human biopsy samples can be examined in Ussing chambers [5, 17]. The left side of the human colon has lower TER than the right (Fig. 7a). This variation has not been shown previously in humans and replicates regional changes observed in mice (Fig. 4a). Mucosal biopsies obtained from a lesion site (within 1 cm of an adenomatous polyp or tumour tissue) showed considerably reduced TER in comparison to samples from healthy individuals (Fig. 7b). There is a higher flux of the paracellular probe lucifer yellow in left sided colonic biopsies compared to right sided samples from healthy patients (Fig. 7c). Lucifer yellow provided a more sensitive measurement of paracellular permeability than 4 kDa FITC-dextran due to its smaller size of 457 Da. Lucifer yellow flux was notably higher in lesion sites when compared to biopsies from the right colon, but was comparable with a proportion of left colon biopsies (mean = 19.67 μg/ml/cm^2^ vs 15.2 μg/ml/cm^2^, respectively). Additional file 4: Figure S2A and B show there is a significant correlation between lucifer yellow flux and TER in the right colon but not in the left (Additional file 4). This supports the fact that lucifer yellow measures only paracellular flux. TER provides an overall permeability reading, taking into account all routes of passage including those created by ionic pores and upregulation/downregulation of TJ proteins.

## Discussion

This report describes methodology to measure intestinal permeability of mouse and human colonic samples ex vivo using the Ussing chamber system. Intestinal epithelial barrier function is crucial for maintaining a symbiotic relationship between the body and commensal microbiota that reside within it. The ability to measure this function is a useful addition to studying gastrointestinal physiology, pharmacology, and disease. The Ussing system can be adapted in-house to support various tissue sizes and is capable of identifying subtle differences in gut integrity at various regions of the colon, including sections of colon surrounding intestinal abnormalities. Historically surgical samples were used but they may become ischaemic if there are delays in collection and require seromuscular stripping (to remove smooth muscle) +/− indomethacin (inhibits tissue cyclooxygenases to prevent the production of inflammatory prostaglandins). It is far more straightforward to use fresh mucosal biopsies obtained during endoscopy. By using a six-chamber system, sub-optimal biopsies (abnormal PD value or unresponsive to forskolin) can be discounted from analyses and the remaining samples used to calculate integrity. We demonstrate the reliability and consistency of Ussing chamber measurements and importantly describe regional variation in colonic permeability. Nevertheless, differences are suggested by embryological origins of hind vs midgut [30], different patterns of disease distribution seen in IBD [31] and expression patterns of TJ proteins [32].

Optimisation of dissection techniques for both mouse and human tissue is important for viability. In this study, experiments ran for a total of ninety minutes, with forskolin addition at sixty minutes. Lengthier experiments are possible (up to 3 h) but tissue viability has to be demonstrated throughout the experimental procedure. Simultaneous electrical and probe flux measurements provide a detailed description of intestinal integrity. Of note, passage of 4 kDa FITC-dextran and other paracellular probes such as ^51^Cr-EDTA show a linear increase over time [33]. Validation of this Ussing chamber system was achieved through measurements using mouse models of chemically and autoimmune induced inflammation. The data described in this report demonstrates for the first time that depletion of Tregs results in reduction of epithelial barrier function, a key aspect in the generation of colitis [29].

Once Ussing chamber methodology is established there are many experimental applications of the technique, such as measuring how drugs or exogenous cytokines impact on permeability or the effect of the microbiome on intestinal function. Furthermore, it can give insight into differing disease states of the alimentary canal. The methodology described within this report should enable those new to the field to construct an Ussing chamber system and obtain reliable measurements of intestinal permeability.

## Conclusions

The Ussing Chamber system is a useful tool for assessing gut integrity within animal models and humans. However, setup of equipment and methodology can be challenging. This study provides detailed information on Ussing Chamber equipment, experimental procedures and results that validate the technique. Importantly this report provides step-by-step detailed information, therefore it can be utilised by scientists from various sub-specialties.

## Additional files

Additional file 1: **Table S1.** Patient Characteristics. (DOCX 16 kb)

Additional file 2: ARRIVE Guidelines Checklist. (DOCX 658 kb)

Additional file 3: **Figure S1.** TER of colonic regions from male and female untreated FoxP3^DTR^ mice. (A) TER of male and female (A) distal, (B) mid and (C) proximal colonic regions. Mann-Whitney tests used for statistical analyses. Error bars show +/− SEM. (DOCX 82 kb)

Additional file 4: **Figure S2.** Correlation between TER and lucifer yellow flux. (A) Correlation between TER and lucifer yellow in the right side of the colon and (B) the left side of the colon. Spearman’s correlation used for statistical analysis. (DOCX 77 kb)

## Data Availability

Raw data files are available from the corresponding author on request. Patient characteristic details can be found in Additional file 1.

## Abbreviations

Ag/AgCl: Silver/Silver chloride
CF: Cystic Fibrosis
DSS: Dextran sodium sulphate
DTx: Diphtheria toxin
I.P: Intraperitoneal injection
IBD: Inflammatory bowel disease
Isc: Short circuit current
PD: Potential difference
R: Resistance
SPF: Specific pathogen free
TER: Transepithelial resistance
TJ: Tight junctional
Tregs: T regulatory cells
ZO-1: Zonula occluden 1
Ω: Ohms
