# Supplementary material for: The Ussing chamber system for measuring intestinal permeability in health and disease
Source: BMC Gastroenterol. 2019 Jun 20;19:98. doi: 10.1186/s12876-019-1002-4 (PMC6585111; doi:10.1186/s12876-019-1002-4)
Supplement: Supplementary file 1 — Table S1. Patient Characteristics. (DOCX 16 kb) [file 12876_2019_1002_MOESM1_ESM.docx]

|  | Patients | Male | Female |
| --- | --- | --- | --- |
| Number (%) | 17 | 9 (53) | 8 (47) |
| Age (range) | 59 (29-71) | 55 (29-70) | 63.5 (56-71) |
| Adenoma patient number | 2 | 0 | 2 |
| Age |  | - | 65 & 68 |
| Location of adenoma | Caecum | - | - |
|  | Transverse | 0 | 1 |
|  | Sigmoid | - | - |
|  | Rectum | 0 | 1 |
|  |  |  |  |
| CRC Patient number | 1 | 1 | 0 |
| Age | 60 | - | - |
| Tumour Location | Rectum | 1 | 0 |

Additional file 1: Table S1 Patient Characteristics.
