# Supplementary material for: The Ussing chamber system for measuring intestinal permeability in health and disease
Source: BMC Gastroenterol. 2019 Jun 20;19:98. doi: 10.1186/s12876-019-1002-4 (PMC6585111; doi:10.1186/s12876-019-1002-4)

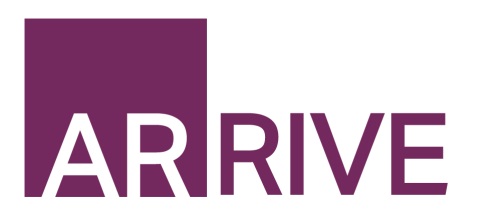


The ARRIVE Guidelines Checklist

Animal Research: Reporting In Vivo Experiments

Carol Kilkenny^1^, William J Browne^2^, Innes C Cuthill^3^, Michael Emerson^4^ and Douglas G Altman^5^

*^1^The National Centre for the Replacement, Refinement and Reduction of Animals in Research, London, UK, ^2^School of Veterinary Science, University of Bristol, Bristol, UK, ^3^School of Biological Sciences, University of Bristol, Bristol, UK, ^4^National Heart and Lung Institute, Imperial College London, UK, ^5^Centre for Statistics in Medicine, University of Oxford, Oxford, UK.*

|  | | ITEM | RECOMMENDATION | Section/ Paragraph |
| --- | --- | --- | --- | --- |
| 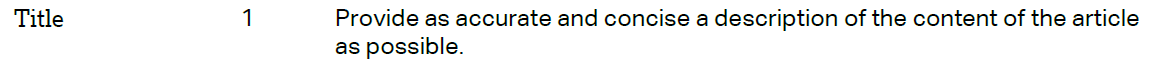 | | | Title |  |
| 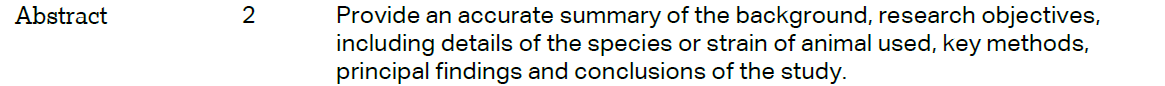 | | | Abstract |  |
| INTRODUCTION | | |  |  |
| 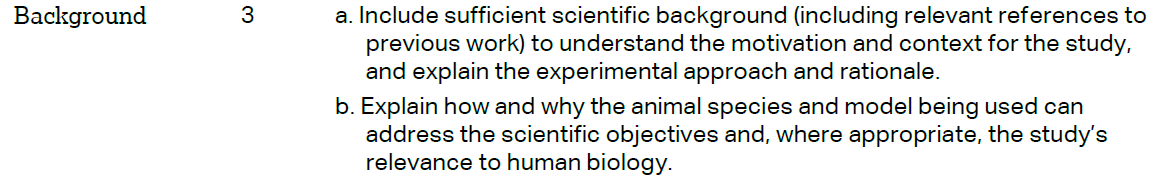 | | | paragraphs 1 -3  paragraph 3 |  |
| 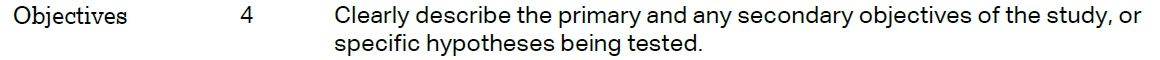 | | | Paragraph 3 |  |
| METHODS | | |  |  |
| 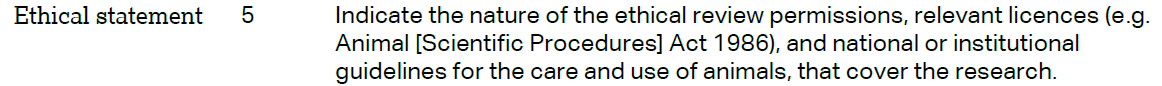 | | | Mice, paragraph 1 |  |
| 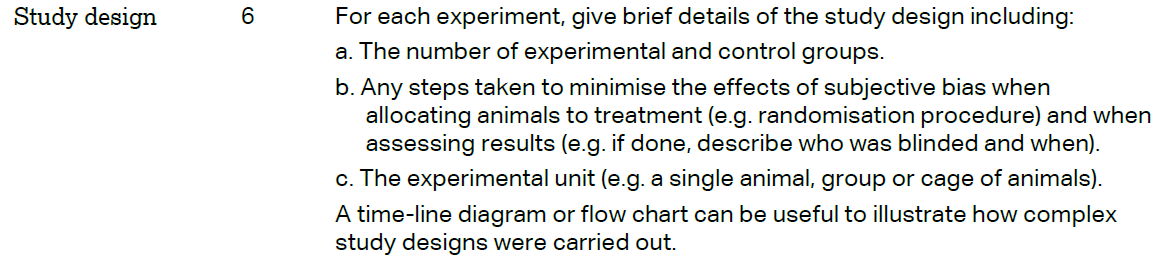 | | | Mice, paragraph 1 and 2  Mice, paragraph 1 and 2  Mice, paragraph 1 and 2 |  |
| 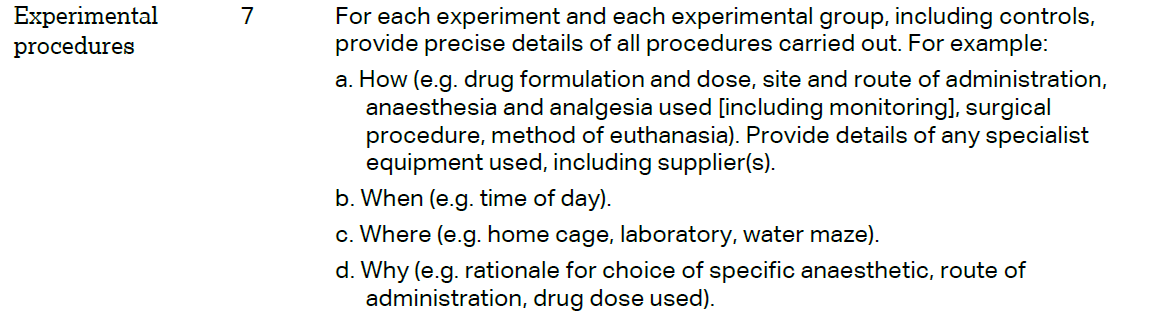 | | | Mice, paragraph 1 and 2  Mice, paragraph 1 and 2  Mice, paragraph 1 |  |
| 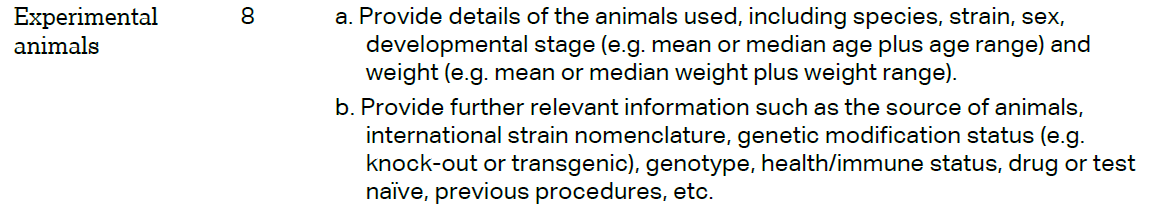 | | | Mice, paragraph 1 and 2  Mice, paragraph 1 and 2 |  |

The ARRIVE guidelines. Originally published in *PLoS Biology*, June 2010^1^

| 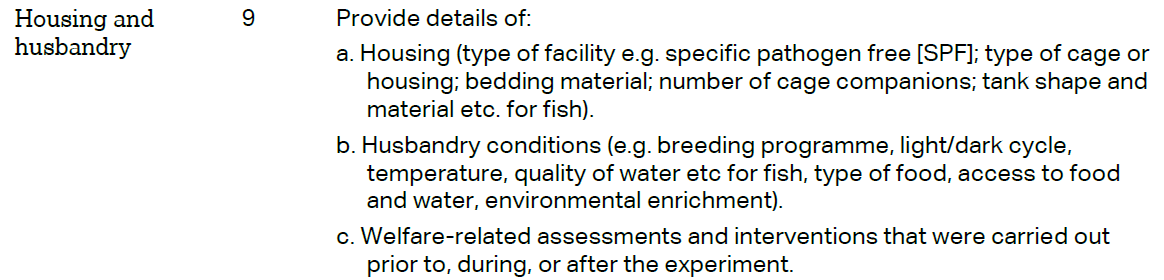 | Mice, paragraph 1  Mice, paragraph 1 and  Mice, paragraph 1 and 2 | |
| --- | --- | --- |
| 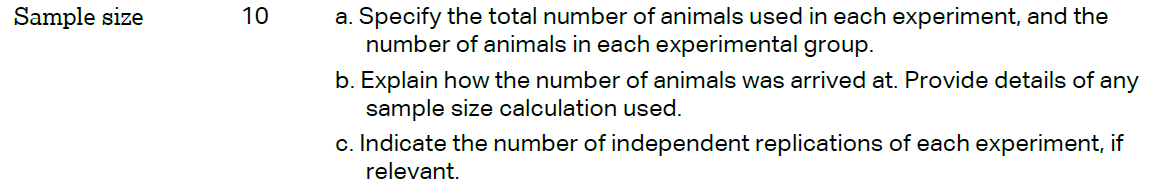 | Mice, paragraph 1 and 2  N/A  Mice, paragraph 2 | |
| 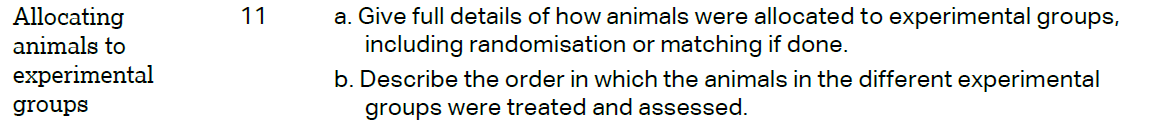 | Mice, paragraph 1 and 2  Mice, paragraph 1 and 2 | |
| 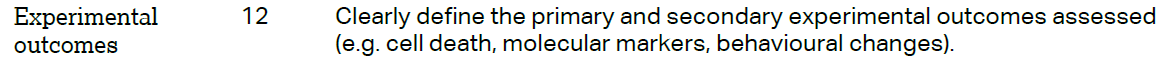 | Mice, paragraph 1 | |
| 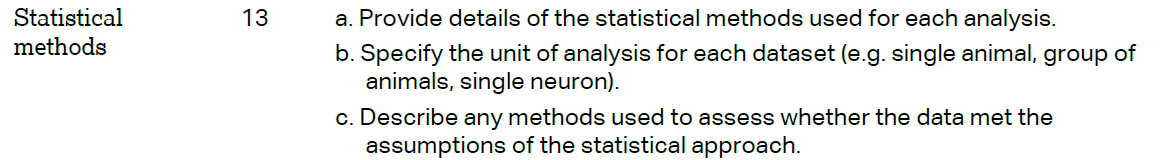 | Statistical analysis  Statistical analysis | |
| RESULTS |  | |
| 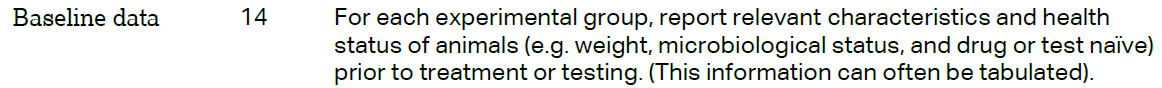 | Mice, paragraph 1 and 2 | |
| 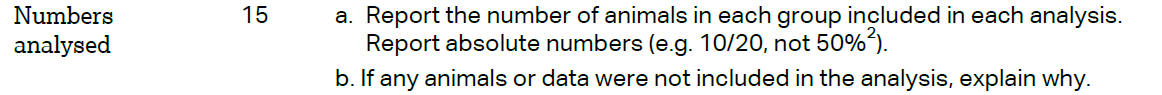 | Mice, paragraph 1 and 2  Electrical measurem-ents, | |
| 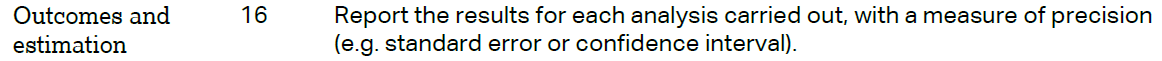 | Paragraphs 1, 2 and 3 | |
| 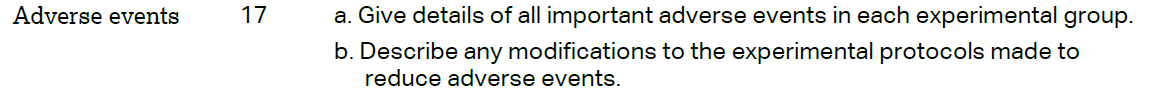 | N/A  N/A | |
| DISCUSSION |  | |
| 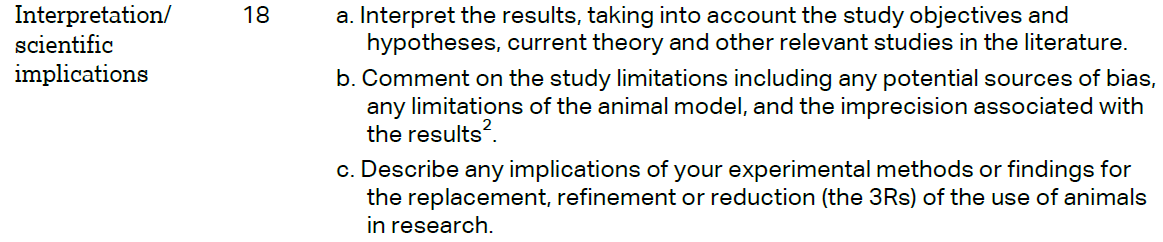 | Paragraphs 1 and 2  N/A  N/A | |
| 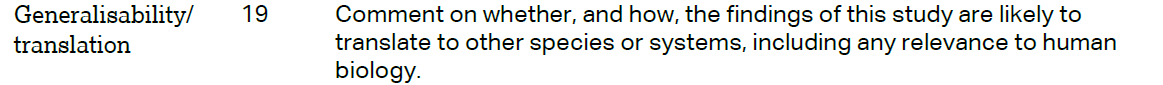 | Paragraph 2 | |
| 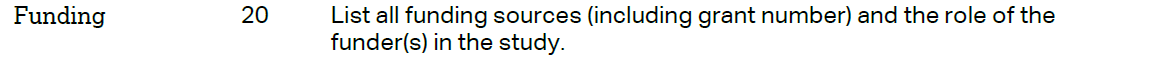 | | Declarations Funding |


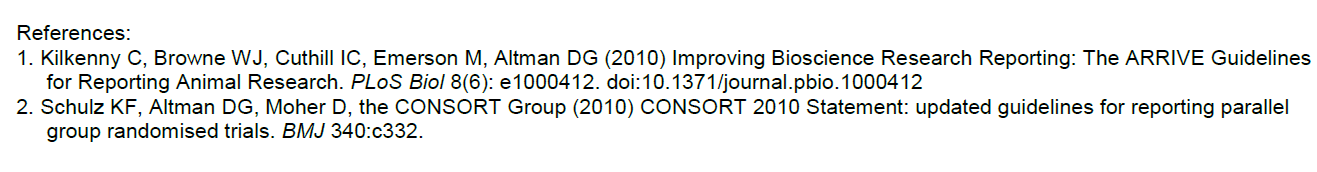

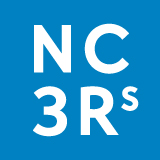

Supplement: Supplementary file 2 — ARRIVE Guidelines Checklist. (DOCX 658 kb) [file 12876_2019_1002_MOESM2_ESM.docx]
