# Supplementary material for: The Ussing chamber system for measuring intestinal permeability in health and disease
Source: BMC Gastroenterol. 2019 Jun 20;19:98. doi: 10.1186/s12876-019-1002-4 (PMC6585111; doi:10.1186/s12876-019-1002-4)
Supplement: Supplementary file 3 — Figure S1. TER of colonic regions from male and female untreated FoxP3DTR mice. (A) TER of male and female (A) distal, (B) mid and (C) proximal colonic regions. Mann-Whitney tests used for statistical analyses. Error bars show +/− SEM. (DOCX 82 kb) [file 12876_2019_1002_MOESM3_ESM.docx]

B

A

C
